# Expensive Treatment

**Published:** 1883-05

**Authors:** 


### EXPENSIVE TREATMENT.

The son of the Grand Duke of one of the North German States, made an engagement for himself and wife with a dentist of his small capital, making the express condition that only new instruments should be used. The surprise of the aristocratic patient knew no limit, when, after an insignificant operation, he was presented with a bill of $750. A celebrated dentist of Berlin, to whom the bill was referred, reported that, in consideration of the requested new instruments, the charges were entirely within reasonable limits, and the young prince paid. But it is said that he has taken an oath to abstain from the luxury of toothache, which was too expensive, even for the income of the son of a Grand Duke.—*Zahntechnische Reform.*

